# Mental Health and Weather Extremes in a Southeastern U.S. City: Exploring Group Differences by Race

**DOI:** 10.3390/ijerph17103411

**Published:** 2020-05-14

**Authors:** Lisa Reyes Mason, Bonita B. Sharma, Jayme E. Walters, Christine C. Ekenga

**Affiliations:** 1 College of Social Work, University of Tennessee, 306 Henson Hall, Knoxville, TN 37996, USA; jwalte22@vols.utk.edu; 2 Department of Social Work, College of Health, Community and Policy, University of Texas at San Antonio, 501 W. Cesar Chavez Blvd., San Antonio, TX 78207, USA; bonita.sharma@utsa.edu; 3 Brown School, Washington University in St. Louis, One Brookings Drive, Campus Box 1196, St. Louis, MO 63130, USA; ekengac@wustl.edu

**Keywords:** mental health, health, weather, climate change, race, heat, cold, summer, winter

## Abstract

The connection between mental health and weather extremes is a public health concern, but less studied to date than physical health. This exploratory study examines the mental health impacts of two kinds of weather extremes increasingly linked to climate change—summer heat waves and extreme winter weather—in a low- to middle-income population in the Southeastern U.S. The distribution of mental health impacts, and potential pathways to them, are examined with a focus on race. Data are from a random-sample survey of 426 participants and are analyzed with bivariate statistics and path analysis. Self-reported mental health impacts, in both seasons, were common in our study, with White participants tending to report worse impacts than participants who identified with other racial groups. Physical health had direct effects on mental health across several models, overall and by racial group. For summer heat waves, concern about climate change and social cohesion had direct and indirect effects, respectively, on mental health in White participants only. For extreme winter weather, preparedness had a direct negative effect on mental health in White, but not Black, participants. Results suggest that there may be racial differences in the influence of human and social capital factors on mental health related to weather extremes, warranting further study of this critical topic and with larger racial subgroup samples.

## 1. Introduction

The global climate crisis is a major threat to public health. The risks of climate change to physical health include increased occurrence of asthma and other respiratory disease, incidence of heat stress, exposure to vector-borne diseases, and injury and fatality during severe weather [1,2]. The effects of climate change on mental health are also concerning though much less studied to date than physical health [3]. Mental health risks of climate change include greater stress, anxiety, despair, depression, and suicidal ideation, and they can stem from climate-related natural disasters (e.g., hurricane), slower moving events (e.g., drought), or concern about the phenomenon of climate change itself [2,4].

In this exploratory study, we focus on the mental health impacts of two kinds of weather extremes increasingly linked to climate change—summer heat waves and extreme winter weather. Informed by a social justice focus on vulnerable or marginalized groups, we examine self-reported mental health impacts among a primarily low- to middle-income population in the Southeastern U.S., and how potential pathways to those impacts vary by race.

### 1.1. Climate Change and Weather Extremes

Attribution science that links specific extreme weather events to climate change is still developing [5]. There is significant evidence, however, that weather extremes are increasing and intensifying in many regions of the world because of human-induced climate change [6,7]. These extremes include worse heat waves in summer and wetter, icier, or colder conditions in winter [6,8].

In June 2019, for example, record heat temperatures were broken in several European countries [9]. In the Southeastern U.S., 61% of major cities are trending toward worsening heat waves, and the average number of nights with temperatures above 75 degrees Fahrenheit nearly doubled the 1901–1960 average during the 2010s [10]. While the number of cold days in many global regions is decreasing with climate change [6], warmer atmospheric conditions and changing Arctic conditions have also been correlated with increased extreme winter weather, such as in the mid-latitudes of the eastern U.S. [11].

Weather extremes impact people in many ways, with often worse consequences for groups with lower social, economic, or political resources, rendering weather and climate issues matters of social justice [12,13]. People who are economically vulnerable, for example, sometimes forego air conditioning or heating during temperature extremes, putting their health at risk out of a fear of higher energy bills and utility disconnections [14,15]. Children and older adults are often particularly susceptible to illness during extreme heat or cold, due to physiological limitations or social isolation [2,16,17]. Weather extremes and climate change may not create these underlying inequalities, but they serve as threat multipliers that can render the quality of life of people who are already vulnerable even worse or more precarious than before [13].

### 1.2. Weather Extremes and Mental Health

While the effects of weather extremes on physical health have been extensively studied [16,18,19], the linkages between such extremes and mental health have only recently emerged as a public health concern [3,20]. These effects on mental health can be direct (e.g., chronic stress as heat waves increase in frequency and severity; post-traumatic stress disorder after a weather-related disaster) or indirect (e.g., increased social isolation during an extreme cold event which then influences depression) [21].

Prior research finds that temperature extremes can increase acute stress as well as worsen mental health problems for people with pre-existing conditions or diagnoses including mood and anxiety disorders [22,23,24]. For high temperatures, effects on mental health were found across gender and education levels among a nationally representative sample in the U.S., with increased vulnerability among older adults and people with less education [25]. Further, studies suggest that some psychotropic medications can affect the brain’s regulation of body temperature, putting people who take these medications at greater risk of illness or even mortality during a heat wave [20].

For low temperatures or winter extremes, icy conditions can lead people to stay at home, temporarily increasing or worsening social isolation, which can be of particular concern for older adults or people with pre-existing depression [4]. If places of employment close during winter extremes, or if people are unable to safely travel to work, lost wages and resulting financial stress can also adversely affect mental health [4]. In addition, since people may be homebound or have few safe transportation options in winter extremes, they may have mental health impacts that they experience on their own (i.e., at home or without seeking professional care) and which are not detected by public health surveillance data that come from health clinic, psychiatric emergency, or hospital admissions data [26].

### 1.3. A Focus on Race

Groups of people often considered more vulnerable to adverse, weather-related, mental health impacts include people with lower incomes, older adults, those already experiencing social isolation, and people with other underlying physical or mental health conditions [2]. One additional kind of social difference that warrants study in this area, but has been relatively less explored, is race.

Particularly in the U.S. context, people from minoritized racial groups may feel that their health is at greater risk from climate change compared to people from the White majority and historically more privileged, powerful group [27]. Yet, there are also questions about whether African Americans, in particular, may have higher coping skills than Whites, a form of resilience in the face of racism and discrimination in their lives [28,29] and which might prove to be a resource in the face of weather-related stress. In our own previous work with the present study’s sample, we found that being non-White, compared to White, was associated with lower levels of self-reported mental health impacts for both summer heat and winter extremes [26]. Additional research on race, mental health, and weather extremes is needed in order to further our understanding of how racial group membership may relate to climate-related impacts.

### 1.4. The Present Study

The present, exploratory study advances our understanding of how weather extremes impact mental health in three important ways. First, it examines more than one kind of extreme with the same population, by looking at the self-reported mental health impacts of summer heat and winter extremes with the same sample. This kind of multihazard analysis is still rare in the climate and weather impacts literature but is necessary to understand how multiple hazards and changing conditions affect people’s lives. Second, it deliberately focuses on a low- to middle-income population, a group often found more vulnerable to the human consequences of climate change than higher income groups, and thus warranting closer study. Third, it looks at how reported mental health impacts, and possible pathways to those impacts, may differ by race.

**Figure 1:**
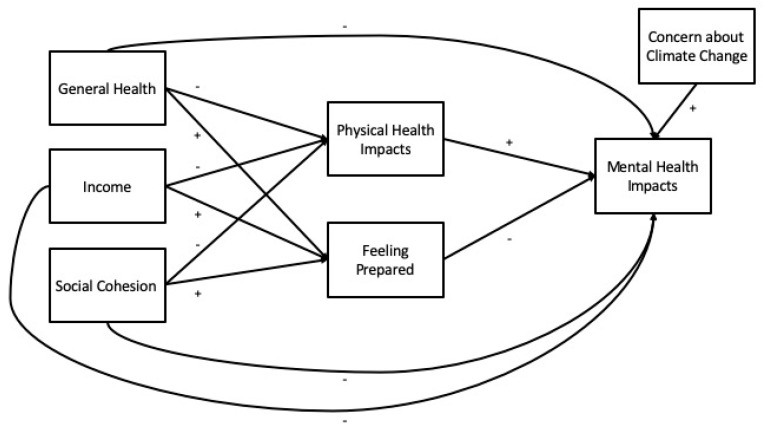
Hypothesized pathways to mental health, with proposed direction of relationships (+/−).

Our analysis of hypothesized pathways (Figure 1) is informed by prior literature on how forms of human capital (e.g., general health, physical health), financial capital (e.g., income), and social capital (e.g., social cohesion) may help people cope with or adapt to the consequences of climate-related weather extremes [20,26,30]. For exploratory purposes, we also include two cognitive variables that may relate to mental health impacts: feeling prepared for extreme weather (preparedness) and concern about climate change.

## 2. Materials and Methods

### 2.1. Survey Sample and Administration

A 56-item questionnaire was mailed to randomly-selected households in Knoxville, Tennessee, a medium-sized city in the Southeastern U.S., in August 2016. Households were in the 25 census tracts in Knoxville, with a median household income at or below $33,494, which was the city median for the period of 2010–2014. One tract that met this study’s income criteria was excluded because it primarily houses undergraduate students. Another tract (which has a growing Hispanic population) was oversampled by a factor of two in order to try and increase the number of responses from Hispanic or Latino individuals in the study. In this tract, surveys were mailed in English and Spanish; in other tracts, only English-language surveys were mailed.

Selected households received an advance postcard, letter and questionnaire, follow-up postcard, and follow-up letter and questionnaire. The letter asked that the adult (age 18 and over) with the most recent birthday complete and return the questionnaire. Upon questionnaire return, participants were sent a $6 gift card. The study response rate was 24.3%, with 442 participants. Given the study’s focus on racial differences, we dropped cases with missing data for race (16% or 3.6% or all participants) for a final sample size of 426 participants. Adjusting for oversampling in one tract, the weighted sample size is 408 participants. This study was approved for ethical conduct of human subjects research by the University of Tennessee Institutional Review Board. Deidentified study data are available via the University of Tennessee TRACE repository.

### 2.2. Measures

#### 2.2.1. Demographic Variables

Participants self-identified their race in response to the question, “What race or races do you consider yourself to be?” They chose one or more from the following options: White or Caucasian, Black or African American, Hispanic or Latino, Asian, American Indian or Alaska Native, and Other (with the option to further specify). We coded responses as 1 = White or Caucasian (hereafter, White), 2 = Black or African American (hereafter, Black), and 3 = American Indian or Alaska Native, Asian, Hispanic or Latino, other as specified by the participant, biracial, or multiracial (hereafter, other or neither White nor Black).

Other demographic variables in this study included gender (female or male), marital status (coded as married or living with a long-term partner, yes or no), and education level (coded as high school diploma or less, some college, or college degree or more).

#### 2.2.2. Endogenous Variables

Endogenous variables in our hypothesized model (Figure 1) are mental health impacts, physical health impacts, and feeling prepared. We measured mental and physical health impacts by asking participants four questions: “To what extent is your [mental/physical] health negatively affected by [very hot temperatures in the summer/extreme winter weather]?” To measure feeling prepared, we asked participants two questions: “In general, how prepared do you feel to stay safe during a heat wave/extreme winter weather?” For each question, participants selected from the following responses: not at all, slightly, somewhat, or very much. We also described “extreme winter weather” as “unusually cold, snowy, or icy conditions in the winter.” For analysis, responses of “somewhat” and “very much” to the two mental health questions were collapsed due to problematic separation (no responses of “very much” for participants who are neither White nor Black). Meanwhile, for the two preparedness questions, responses of “not at all” and “slightly” questions were collapsed based on the data distribution, which had small percentages of participants choosing one of these responses, for either season.

#### 2.2.3. Exogenous Variables

Exogenous variables in our hypothesized model (Figure 1) are general health, income, social cohesion, and concern for climate change. For general health, we asked participants, “How would you rate your general health status?” and provided five response options on a Likert-like scale ranging from very poor to very good. For income, participants chose their annual gross household income from a list of eight options ranging from “less than $10,000” to “$95,000 or more.” Social cohesion was measured with a score of 1 to 5 and using the average of responses (reverse-coded when needed) to the five items on the Social Cohesion and Trust scale [31], e.g., “People around here are willing to help their neighbors” with a 5-point response option from 1 = strongly disagree to 5 = strongly agree). To measure concern for climate change, we asked participants, “To what extent do you feel concerned about climate change in Knoxville?” Participants selected from the following responses: not at all, slightly, somewhat, or very much. For analysis, we treated general health, income, social cohesion, and concern for climate change as continuous.

### 2.3. Analyses

We conducted descriptive statistics and bivariate analyses by race with SPSS 24.0. We then conducted path analyses using weighted least squares mean and variance-adjusted (WLSMV) estimation with bootstrapped (*n* = 1000) confidence intervals for standardized estimates with Mplus 8.1 [32]. For the summer analyses, we ran a full model and subgroup models for the three racial groups: White, Black, and other. For the winter analyses, standard errors of parameter estimates could not be computed for the racial group of “other;” thus, we included only White and Black participants in these analyses. We used the “model indirect” command in Mplus to assess for indirect effects, which computes standard errors for these effects through the Delta method [33].

We assessed model fit with multiple criteria [34,35,36]. For absolute or predictive fit, we report the chi-square (*X*^2^) test of model fit along with the comparative fit index (good ≥ 0.95). The standardized root mean square residual (good ≤ 0.08) and root mean square error of approximation (good ≤ 0.05) are also reported. We also assessed the suitability of using the same exploratory model across racial subgroups through generation and review of modification indices, which were set at a minimum value of 10 to control for a Type I error.

## 3. Results

### 3.1. Sample Characteristics and Descriptives

Demographic characteristics, weighted for oversampling in one census tract, are presented in Table 1. Overall, our sample had a higher percentage of White (72.7% vs. 66.0%), female (64.1% vs. 52.3%), and college-educated or higher (35.0% vs. 20.1%) participants than the sampled census tracts as a whole (based on 2015 US Census Bureau data). Bivariate differences by race were found for marital status (*X*^2^(2, *N* = 405) = 8.72, *p* = 0.013) and education level (*X*^2^(4, *N* = 406) = 20.94, *p* = 0.000). When demographic variables were controlled for in path analyses, results did not substantially differ; thus, they were omitted from final path models.

Descriptives for endogenous and exogenous variables are presented in Table 2 and Table 3, along with statistically significant bivariate associations for these variables with race. Overall, participants reported similar levels of mental health impacts whether for summer or winter extremes (56.4% reporting any degree of impact for summer, 52.2% for winter). Physical health impacts were reported to a greater extent than mental health impacts overall and were worse for summer than winter (77.4% reporting any degree of impact, compared to 65.7%).

Statistically significant differences by race were found for mental health impacts in summer (*X*^2^(4, *N* = 399) = 14.12, *p* = 0.007) and winter (*X*^2^(4, *N* = 399) = 19.24, *p* = 0.001), with White participants tending to report worse impacts among the three racial groups studied. For summer, 61.8% of White participants reported any mental health impact, compared to 41.0% of Black participants and 46.4% of participants who are neither White nor Black. For winter, 58.9% of White participants reported any mental health impact, compared to 34.5% of Black participants and 35.8% of participants who are neither White nor Black.

A statistically significant difference by race was found for income (*F*(2, *N* = 391) = 7.27, *p* = 0.001). Post-hoc analyses found that mean income for participants in the Black racial group (2.60) was lower than for those in the White (3.51) or other (3.62) racial group.

### 3.2. Path Analyses

Model fit statistics are summarized in Table 4. The summer heat models suggest an excellent fit to the data, and the winter extreme models suggest a good fit.

#### 3.2.1. Summer Heat, Overall

**Figure 2:**
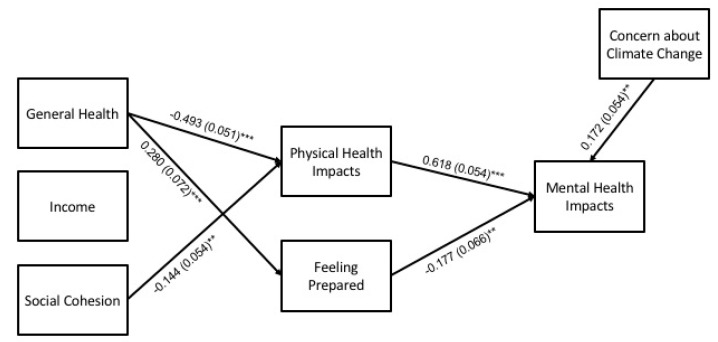
Path model for mental health impacts during summer heat (N = 426). Statistically significant pathways are shown with standardized estimates and standard errors (SEs) in parentheses. Statistical significance levels are: ** *p* < 0.01, and *** *p* < 0.001.

For the overall summer model (N = 426), hypothesized pathways with empirical support are shown in Figure 2. In this and subsequent figures, solid lines are used for pathways with statistical significance at *p* < 0.05, and dashed lines for pathways with statistical significance at *p* < 0.10 given the exploratory nature of the study and small sample size of two of the racial groups, which limits statistical power. Full results for the overall model are detailed in Table 5. Statistically significant pathways (at *p* < 0.05) and key findings are summarized in the following paragraphs. 

**Direct effects**. General health and social cohesion were negatively associated with physical health impacts, and general health was positively associated with feeling prepared. Concern about climate change and physical health impacts were positively associated with mental health impacts, and feeling prepared was negatively associated.

**Indirect effects**. The model showed statistically significant indirect effects of general health and social cohesion on levels of mental health impacts through levels of physical health impacts. General health also had a statistically significant indirect effect on mental health impacts through feeling prepared.

#### 3.2.2. Summer Heat, Subgroups

**Figure 3:**
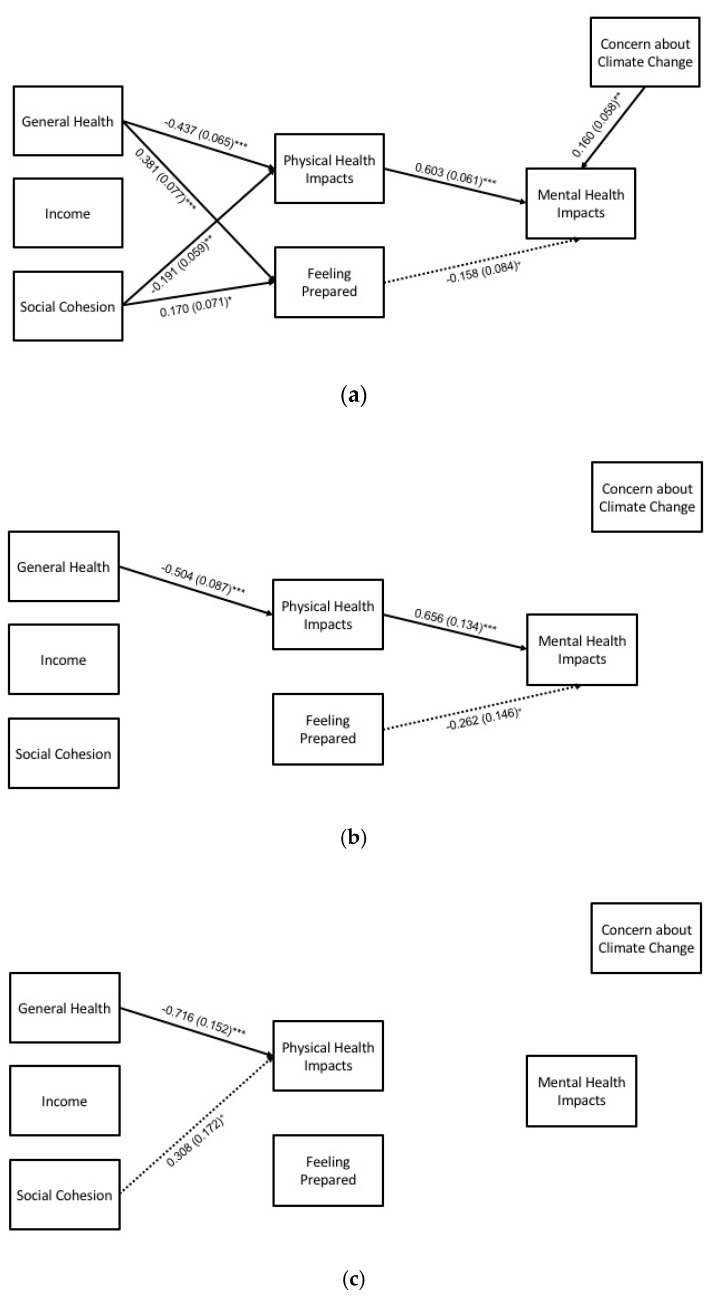
Path model for mental health impacts during summer heat (N = 426), by racial subgroup: (**a**) White (*n* = 308), (**b**) Black (*n* = 89), and (**c**) other (*n* = 29) participants. Statistically significant pathways are shown with standardized estimates and standard errors (SEs) in parentheses. Statistical significance levels are: ^+^ *p* < 0.10, * *p* < 0.05, ** *p* < 0.01, and *** *p* < 0.001.

For the summer subgroup analyses, hypothesized pathways with empirical support are shown in Figure 3, and full results are detailed in Table 6. Key findings for statistically significant pathways (at *p* < 0.05) for each group are summarized in the following paragraphs. 

*Direct effects.* General health was negatively associated with physical health impacts for all three subgroups. For White participants, social cohesion was negatively associated with physical health impacts, and general health and social cohesion were positively associated with feeling prepared. Physical health impacts were positively associated with mental health impacts for the Black and White subgroups. Concern about climate change was positively associated with mental health impacts for the White subgroup.

**Indirect effects**. General health had an indirect effect on mental health impacts through physical health impacts for the Black and White subgroups. For the White subgroup, social cohesion had an indirect effect on mental health impacts through physical health impacts. No indirect effects were found for the “other” subgroup.

#### 3.2.3. Winter Extremes, Overall

**Figure 4:**
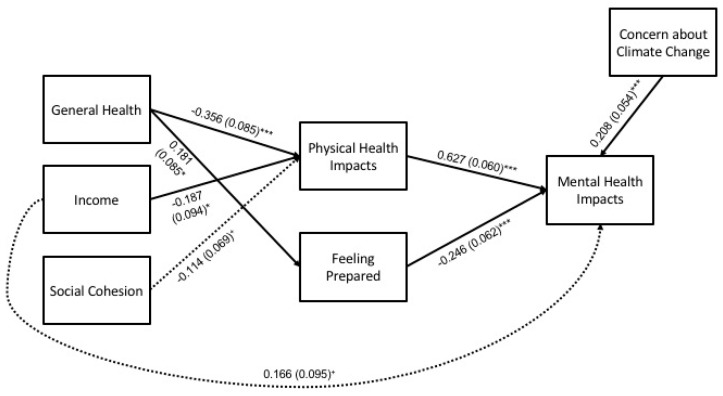
Path model for mental health impacts during winter extremes (N = 397). Statistically significant pathways are shown with standardized estimates and standard errors (SEs) in parentheses. Statistical significance levels are: ^+^ *p* < 0.10, * *p* < 0.05, and *** *p* < 0.001.

For the overall winter model (*N* = 397), hypothesized pathways with empirical support are shown in Figure 4, and full results are detailed in Table 7. Statistically significant pathways (at *p* < 0.05) and key findings are summarized in the following paragraphs.

**Direct effects**. General health and income were negatively associated with physical health impacts. General health was positively associated with feeling prepared. Related to mental health impacts, concern for climate change and physical health impacts had positive associations, while feeling prepared had a negative one. 

**Indirect effects**. Only one indirect effect was detected; general health had an indirect effect on mental health impacts through physical health impacts.

#### 3.2.4. Winter Extremes, Subgroups

**Figure 5:**
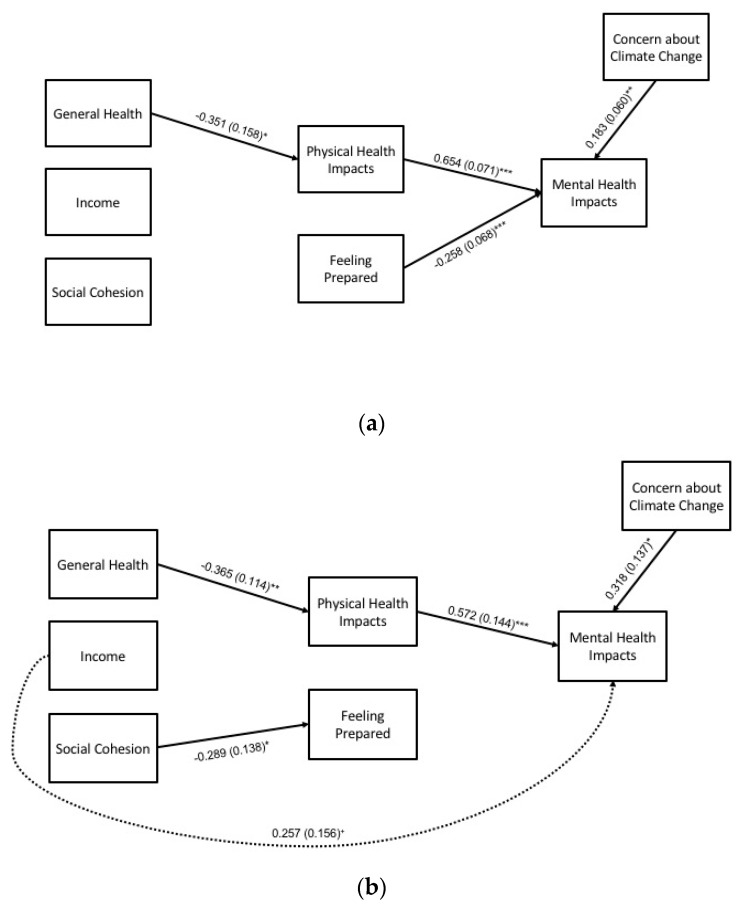
Path model for mental health impacts during winter extremes (*N* = 397), by racial subgroup: (**a**) White (*n* = 308), and (**b**) Black (*n* = 89) participants. Statistically significant pathways are shown with standardized estimates and standard errors (SEs) in parentheses. Statistical significance levels are: ^+^ *p* < 0.10, * *p* < 0.05, ** *p* < 0.01, and *** *p* < 0.001.

For the winter subgroup analyses, hypothesized pathways with empirical support are shown in Figure 5, and full results are detailed in Table 8. Key findings for statistically significant pathways (at *p* < 0.05) for each group are summarized in the following paragraphs.

*Direct effects*. For both the Black and White subgroups, general health was negatively associated with physical health impacts. Social cohesion was negatively associated with feeling prepared for the Black subgroup. Concern about climate change and physical health impacts had positive associations with mental health impacts for both subgroups, while feeling prepared was negatively associated with mental health impacts for the White subgroup only.

*Indirect effects*. As in the overall model, general health had an indirect effect on mental health impacts through physical health impacts for both subgroups.

## 4. Discussion

This exploratory study examined the mental health impacts of two kinds of weather extremes increasingly linked to climate change—summer heat waves and extreme winter weather—in a low- to middle-income population in the Southeastern U.S. We used path analysis to explore whether human capital such as general and physical health status, social capital, extreme weather preparedness, and concern about climate change influenced self-reported mental health.

Overall, we observed that mental health during weather extremes was influenced by physical health directly and general health indirectly, irrespective of race or type of weather extreme. We also observed mediating relationships between human and social capital that may explain racial differences in the mental health impacts of extreme weather events, though our results should be interpreted cautiously in light of small subsample sizes for the non-White groups in particular and due to measurement limitations (Section 4.7). We discuss direct and indirect findings in the context of previous research and implications for public health preparedness.

### 4.1. Race and Mental Health Impacts

For both summer heat and winter extremes, a statistically significant higher proportion of White participants reported any mental health impact than Black participants and those who were neither White nor Black. Our findings are consistent with studies in general settings in the U.S. that show people from racial minority groups reporting lower rates of mental health disorders than Whites [37,38]. It is possible that factors such as greater resilience and coping behaviors in the face of stressful situations account for the relatively better outcomes among racial minority groups [39,40]. At the same time, it may be that other societal challenges (e.g., racism, violence) are felt as a more pressing priority or stronger influence on mental health and well-being, for racial minority groups, than environmental issues [41], such as the weather extremes in this study.

Although Black and other racial minority participants in our study reported lower rates of mental health impacts than White participants, their elevated rates overall (41–46% during summer heat and 35–36% during winter extremes) of these outcomes are a concern. In the U.S., racial minorities are more likely to report higher rates of psychological distress than Whites [38], and when diagnosed with mental health disorders, they are more likely to experience more severe and persistent episodes than Whites [37,38,42]. This is of great public health concern as there may be racial disparities in the long-term need for mental health care after extreme weather events, yet access to mental health is also a greater challenge in the U.S. for people who are non-White than White [43].

### 4.2. Direct Effects of Physical Health Impacts

Physical health impacts was the only factor that had a direct effect on mental health impacts in each path model across all racial groups, with the exception of the summer heat analysis for participants in the “neither White nor Black” group, though this latter analysis was limited by its small subgroup sample size. Although a positive relationship between physical health and mental health has been well documented, the causal mechanism is unknown. In an extreme weather event, a physical impact could initiate a pathophysiological response that influences psychological functioning, thus leading to adverse mental health impacts. Additionally, physical health challenges in and of themselves could create stressful conditions, also increasing the likelihood of adverse mental health impacts. Several longitudinal studies in general settings have provided evidence of poor physical health as a risk factor for poor mental health [44,45,46]; however, few studies, to date, have focused on relationships between physical and mental health impacts during summer heat and winter extremes. If longitudinal associations between physical health impacts and subsequent mental health impacts are confirmed in extreme weather settings, promoting mental well-being in conjunction with physical well-being during and after an extreme weather event may be an important approach to consider when developing strategies to reduce the risk of adverse health outcomes. Emergency and urgent care systems, for example, could implement a rapid mental health assessment to identify those with critical mental needs, and long-term follow-up could be implemented to inform the delivery of clinical services.

### 4.3. Concern about Climate Change

Each of the three racial groups in our study had similar levels of concern about climate change. These results contrast with those of other U.S. studies that report racial/ethnic disparities in concern about climate change, with non-White minorities reporting higher levels of concern than Whites [47,48,49]. We found that concern about climate change was directly positively associated with mental health impacts, overall and for both White and Black participants in winter extremes. We also found that, although concern about climate change was directly positively associated with mental health impacts among White participants during summer heat events, there was no association between concern about climate change and mental health impacts among Black participants, though, again, we caution interpretation in light of our subgroup sample sizes. Given that residents of the Southeastern U.S. are expected to experience more frequent climate-related heat events in the future [10], a better understanding of how concern about climate change may affect the mental health of this geographically vulnerable population is necessary. One possible explanation is that mental distress may result from a perceived inability to impact the global scale of climate change, which may trigger a sense of helplessness [50]. Further research on this, with differentiation among social groups such as by race and with larger subgroup samples, is recommended.

### 4.4. Extreme Weather Preparedness

We examined the mental health impact of extreme weather preparedness on mental health and observed direct effects during both summer heat and winter extremes. The direct effects of preparedness on winter-related mental health impacts differed by race. For winter extremes only, we observed a direct negative association between preparedness and mental health among White, but not Black, participants. Preparedness is a key intervention for ensuring effective responses to climate-related hazards [51], and our finding supports an additional role for preparedness as a factor that is protective against adverse mental health impacts. Further, future research should consider what preparedness means for different groups and how it is developed, given the generally lower income of Black than White participants in this study and how preparedness for weather extremes may rank in comparison to other priorities of everyday life [41].

### 4.5. Indirect Effects of General Health

The results of our study suggest that health-related forms of human capital may help protect people from the mental health consequences of weather extremes. For example, for both summer heat and winter extremes, the mental health impact of general health was mediated through physical health impacts in both the White and Black participant groups. In theory, this means that the degree to which a participant’s general health influenced their mental health depended on impacts to their physical health, again indicating that both physical and mental health needs should be addressed when providing services during and after an extreme weather event.

### 4.6. Race and the Indirect Effects of Social Cohesion

Social cohesion was the only variable for which we found a racial difference in its indirect effect on mental health, though the caution made previously about subgroup sample size applies. We observed this difference for summer heat, for which social cohesion indirectly affected mental health through preparedness for White, but not Black, participants. Our findings for White participants provide support for the hypothesized role of social capital in extreme weather adaptation, although there may have been unmeasured variables that influence both social cohesion and preparedness. Few studies have examined these factors during both summer heat and winter extremes, and examining the indirect effects of social capital, and how they vary between racial groups, will be important for planning a public mental health response to climate-related weather extremes.

### 4.7. Limitations

The cross-sectional design of our study limited our interpretation of the relationships between human capital, social capital, extreme weather preparedness, concern about climate change, and subsequent mental health impacts. To fully address causality, a longitudinal study design should be used in future investigations. Self-reported health measures were also a limitation of this study. Our single-item measure of mental health, in particular, was unlikely to capture the full range of mental health impacts that result from weather extremes. Further, a timeframe for the mental health measures was not specified; some participants may have responded with a particular event in mind, while others may have thought across events more generally. Finally, despite our random sampling approach, our study was not fully representative of the sampled census tracts as a whole, the relatively small number of non-Black minorities limited some analyses to White and Black participants only, and the relatively small subgroup samples for Black and other participants limited statistical power to detect associations among variables for these subgroups that may actually exist. Given these limitations, it will be important to replicate this work with larger samples, more refined measures of mental health, and among different climate areas with racially diverse populations.

## 5. Conclusions

In summary, self-reported levels of mental health impacts during weather extremes were common overall and higher among White participants than Black and other racial minority participants in this study, though results should be interpreted cautiously due to small samples of the latter two groups. We found that there were forms of human and social capital that may influence mental health during extreme weather events, and that the influences of these factors varied by race, with mediating effects observed more so for White participants than those who identified with other racial groups in this exploratory study. If confirmed in longitudinal studies and ones with larger racial subgroup samples, the results of this study suggest that communities and practitioners should account for the complex needs of diverse populations when developing engagement strategies to address the mental health impacts of climate-related weather extremes.

## Figures and Tables

**Table 1:** Demographic characteristics, weighted (*N* = 408).

Characteristic	White (*n* = 297)	Black (*n* = 84)	Other (*n* = 28)	All (*N* = 408)
Gender, female (%)	62.6	72.6	53.6	64.1
Married or living with partner (%) ^*^	40.0	25.6	53.6	38.0
Education level (%) ^***^				
High school diploma or less	29.5	44.6	21.4	32.0
Some college ^1^	29.8	37.3	53.6	33.0
College degree or more	40.7	18.1	25.0	35.0

^1^ Includes technical or associate’s degree. ^*^ Bivariate association at *p* < 0.05. ^***^ Bivariate association at *p* < 0.001.

**Table 2:** Endogenous variables by racial group, weighted (N = 408).

Variables	White (*n* = 297)	Black (*n* = 84)	Other (*n* = 28)	All (*N* = 408)
Mental health impacts, summer (%) ^**^				
Not at all	38.2	59.0	53.6	43.6
Slightly	31.9	19.3	32.1	29.3
Somewhat or very much	29.9	21.7	14.3	27.1
Mental health impacts, winter (%) ^**^				
Not at all	41.1	65.5	64.3	47.9
Slightly	31.4	15.5	17.9	27.1
Somewhat or very much	27.5	19.0	17.9	25.1
Physical health impacts, summer (%)				
Not at all	20.3	23.8	41.4	22.6
Slightly	31.4	28.6	27.6	30.5
Somewhat	32.1	28.6	13.8	30.0
Very much	16.2	19.0	17.2	16.9
Physical health impacts, winter (%)				
Not at all	31.7	39.5	44.8	34.3
Slightly	33.1	19.8	24.1	29.8
Somewhat	24.1	24.7	24.1	24.3
Very much	11.0	16.0	6.9	11.8
Feels prepared, summer (%)				
Not at all or slightly	7.7	7.3	18.5	8.4
Somewhat	40.9	42.7	37.0	41.0
Very much	51.4	50.0	44.4	50.6
Feels prepared, winter (%)				
Not at all or slightly	11.7	4.8	17.9	10.7
Somewhat	48.1	53.0	46.4	49.0
Very much	40.2	42.2	35.7	40.3

^**^ Bivariate association at *p* < 0.01.

**Table 3:** Exogenous variables by racial group, weighted (*N* = 408).

Variables	White (*n* = 297)	Black (*n* = 84)	Other (*n* = 28)	All (*N* = 408)
General health (mean (SD ^1^)) ^2^	3.8 (1.0)	3.7 (0.8)	3.8 (1.2)	3.8 (1.0)
Income (mean (SD)) ^3,*^	3.5 (2.1)	2.6 (1.3)	3.6 (2.0)	3.3 (2.0)
Social cohesion (mean (SD))	3.4 (0.9)	3.3 (0.8)	3.3 (0.8)	3.4 (0.9)
Concern for climate change (%)				
Not at all	17.8	15.9	17.9	17.4
Slightly	18.5	25.6	17.9	19.9
Somewhat	38.0	40.2	35.7	38.3
Very much	25.7	18.3	28.6	24.4

^1^ SD = standard deviation. ^2^ 3 = Neither good nor poor; 4 = Good. ^3^ 2 = $10,000 to <$20,000; 3 = $20,000 to <$35,000; 4 = $35,000 to <$50,000. ^*^ Bivariate association at *p*<.05.

**Table 4:** Model fit statistics for path analyses ^1^.

Model	*X*^2^ (df, *p*)	RMSEA (CI)	CFI	SRMR
Summer, overall	6.107 (3, 0.107)	0.049 (0.000–0.106)	0.993	0.016
Summer, subgroups	9.812 (9, 0.366)	0.025 (0.000–0.100)	0.998	0.021
Winter, overall	15.183 (3, 0.002)	0.101 (0.055–0.154)	0.973	0.030
Winter, subgroups	16.208 (6, 0.013)	0.093 (0.039–0.148)	0.979	0.030

^1^*X*^2^ = chi-square; df = degrees of freedom; *p* = *p*-value; RMSEA = root mean square error of approximation; CI = 90% confidence interval; CFI = comparative fit index; SRMR = standardized root mean square residual.

**Table 5:** Path analysis results for summer heat, overall (N = 426).^1^

Path	*β*	95L	95U	SE	*p*
*Direct effects*					
Gen. health → PHI	−0.493	−0.600	−0.398	0.051	0.000
Income → PHI	−0.037	−0.145	0.094	0.062	0.559
Soc. cohes. → PHI	−0.144	−0.258	−0.042	0.054	0.008
Gen. health → Feel. prep.	0.280	0.129	0.426	0.072	0.000
Income → Feel. prep.	0.048	−0.092	0.192	0.076	0.528
Soc. cohes. → Feel. prep.	0.098	−0.017	0.221	0.061	0.104
Concern CC → MHI	0.172	0.066	0.270	0.054	0.001
PH impacts → MHI	0.618	0.512	0.725	0.054	0.000
Feel. prep. → MHI	−0.177	−0.311	−0.052	0.066	0.007
Gen. health → MHI	0.114	−0.032	0.258	0.073	0.122
Income → MHI	0.057	−0.065	0.171	0.060	0.343
Soc. cohesion → MHI	−0.047	−0.154	0.060	0.055	0.399
*Indirect effects*					
Gen. health → PHI → MHI	−0.305	−0.402	−0.230	0.044	0.000
Gen. health → Feel. prep. → MHI	−0.050	−0.108	−0.012	0.024	0.040
Income → PHI → MHI	−0.023	−0.094	0.058	0.039	0.563
Income → Feel. prep. → MHI	−0.008	−0.041	0.018	0.015	0.569
Soc. cohes. → PHI → MHI	−0.089	−0.163	−0.026	0.034	0.009
Soc. cohes. → Feel. prep. → MHI	−0.017	−0.050	0.003	0.013	0.192

^1^*β* = standardized estimate; 95L = lower bound of the bootstrapped 95% *β* confidence interval; 95U = upper bound of the bootstrapped 95% *β* confidence interval; SE = standard error; *p* = *p*-value; PHI = physical health impacts; MHI = mental health impacts; CC = climate change.

**Table 6:** Path analysis results for summer heat, by subgroup (N = 426).^1.^

	White (*n* = 308)					Black (*n* = 89)					Other (*n* = 29)				
Path	*β*	95L	95U	SE	*p*	*β*	95L	95U	SE	*p*	*β*	95L	95U	SE	*p*
**Direct effects**															
Gen. health → PHI	−0.437	−0.561	−0.315	0.065	0.000	−0.504	−0.673	−0.336	0.087	0.000	−0.716	−0.977	−0.412	0.152	0.000
Income → PHI	−0.042	−0.192	0.111	0.076	0.581	−0.083	−0.314	0.134	0.110	0.449	−0.241	−0.657	0.166	0.231	0.297
Soc. cohes. → PHI	−0.191	−0.304	−0.075	0.059	0.001	−0.164	−0.399	0.105	0.129	0.203	0.308	−0.031	0.611	0.172	0.074
Gen. health → Feel. prep.	0.381	0.235	0.540	0.077	0.000	0.049	−0.261	0.313	0.154	0.749	−0.087	−0.645	0.615	0.367	0.813
Income → Feel. prep.	−0.014	−0.212	0.159	0.095	0.880	−0.013	−0.298	0.278	0.155	0.932	0.353	−0.404	1.083	0.410	0.389
Soc. cohes. → Feel. prep.	0.170	0.024	0.305	0.071	0.016	−0.115	−0.447	0.160	0.148	0.437	−0.066	−0.760	0.494	0.333	0.843
Concern CC → MHI	0.160	0.038	0.269	0.058	0.006	0.190	−0.127	0.482	0.149	0.202	0.355	−0.134	1.137	0.344	0.303
PH impacts → MHI	0.603	0.480	0.723	0.061	0.000	0.656	0.370	0.896	0.134	0.000	1.007	−0.889	2.064	0.683	0.141
Feel. prep. → MHI	−0.158	−0.316	0.033	0.084	0.059	−0.262	−0.519	0.039	0.146	0.072	−0.213	−1.059	0.368	0.363	0.558
Gen. health → MHI	0.076	−0.085	0.248	0.090	0.401	0.165	−0.099	0.471	0.158	0.294	0.566	−1.000	2.008	0.810	0.485
Income → MHI	0.022	−0.108	0.158	0.068	0.743	0.125	−0.148	0.459	0.163	0.443	0.311	−1.418	1.183	0.600	0.604
Soc. cohesion → MHI	−0.058	−0.170	0.083	0.066	0.374	0.007	−0.350	0.280	0.152	0.962	−0.297	−0.890	0.535	0.394	0.451
**Indirect effects**															
Gen. health → PHI → MHI	−0.263	−0.375	−0.172	0.051	0.000	−0.331	−0.515	−0.178	0.086	0.000	−0.721	−1.491	0.681	0.457	0.114
Gen. health → Feel. prep. → MHI	−0.060	−0.141	0.011	0.036	0.098	−0.013	−0.120	0.089	0.051	0.802	0.019	−0.256	0.509	0.197	0.925
Income → PHI → MHI	−0.025	−0.114	0.068	0.047	0.588	−0.054	−0.234	0.086	0.077	0.478	−0.242	−0.975	0.270	0.355	0.494
Income → Feel. prep. → MHI	0.002	−0.031	0.047	0.017	0.895	0.003	−0.120	0.082	0.051	0.946	−0.075	−0.893	0.281	0.308	0.807
Soc. cohes. → PHI → MHI	−0.115	−0.189	−0.044	0.037	0.002	−0.108	−0.297	0.071	0.092	0.243	0.310	−0.285	1.048	0.346	0.370
Soc. cohes. → Feel. prep. → MHI	−0.027	−0.075	0.004	0.020	0.171	0.030	−0.044	0.169	0.052	0.563	0.014	−0.395	0.333	0.158	0.929

^1.^*β* = standardized estimate; 95L = lower bound of the bootstrapped 95% *β* confidence interval; 95U = upper bound of the bootstrapped 95% *β* confidence interval; SE = standard error; *p* = *p*-value; PHI = physical health impacts; MHI = mental health impacts; CC = climate change.

**Table 7:** Path analysis results for winter extremes, overall (*N* = 397).^1^

Path	*β*	95L	95U	SE	*p*
*Direct effects*					
Gen. health → PHI	−0.356	−0.500	−0.211	0.085	0.000
Income → PHI	−0.187	−0.339	−0.019	0.094	0.046
Soc. cohes. → PHI	−0.114	−0.242	0.018	0.069	0.097
Gen. health → Feel. prep.	0.181	0.043	0.325	0.085	0.034
Income → Feel. prep.	0.132	−0.042	0.296	0.096	0.172
Soc. cohes. → Feel. prep.	0.027	−0.117	0.171	0.071	0.702
Concern CC → MHI	0.208	0.101	0.312	0.054	0.000
PH impacts → MHI	0.627	0.512	0.748	0.060	0.000
Feel. prep. → MHI	−0.246	−0.371	−0.131	0.062	0.000
Gen. health → MHI	−0.012	−0.155	0.159	0.092	0.893
Income → MHI	0.166	0.015	0.324	0.095	0.081
Soc. cohesion → MHI	−0.028	−0.171	0.100	0.067	0.672
*Indirect effects*					
Gen. health → PHI → MHI	−0.223	−0.341	−0.132	0.066	0.001
Gen. health → Feel. prep. → MHI	−0.044	−0.098	−0.007	0.028	0.110
Income → PHI → MHI	−0.117	−0.230	−0.012	0.070	0.092
Income → Feel. prep. → MHI	−0.032	−0.089	0.009	0.030	0.279
Soc. cohes. → PHI → MHI	−0.072	−0.160	0.011	0.045	0.113
Soc. cohes. → Feel. prep. → MHI	−0.007	−0.043	0.032	0.018	0.710

^1^*β* = standardized estimate; 95L = lower bound of the bootstrapped 95% *β* confidence interval; 95U = upper bound of the bootstrapped 95% *β* confidence interval; SE = standard error; *p* = *p*-value; PHI = physical health impacts; MHI = mental health impacts; CC = climate change.

**Table 8:** Path analysis results for winter extremes, by subgroup (N = 397).^1.^

	White (*n* = 308)					Black (*n* = 89)				
Path	*β*	95L	95U	SE	*p*	*β*	95L	95U	SE	*p*
**Direct effects**										
Gen. health → PHI	−0.351	−0.621	−0.165	0.158	0.026	−0.365	−0.571	−0.133	0.114	0.001
Income → PHI	−0.212	−0.430	0.127	0.189	0.263	−0.121	−0.368	0.150	0.134	0.365
Soc. cohes. → PHI	−0.129	−0.312	0.030	0.093	0.166	−0.076	−0.354	0.200	0.147	0.606
Gen. health → Feel. prep.	0.192	−0.005	0.442	0.137	0.161	0.095	−0.224	0.396	0.158	0.546
Income → Feel. prep.	0.130	−0.184	0.362	0.163	0.425	0.052	−0.207	0.339	0.139	0.710
Soc. cohes. → Feel. prep.	0.102	−0.069	0.277	0.091	0.265	−0.289	−0.578	−0.026	0.138	0.036
Concern CC → MHI	0.183	0.060	0.298	0.060	0.002	0.318	0.064	0.593	0.137	0.020
PH impacts → MHI	0.654	0.524	0.792	0.071	0.000	0.572	0.314	0.883	0.144	0.000
Feel. prep. → MHI	−0.258	−0.402	−0.135	0.068	0.000	−0.145	−0.500	0.189	0.175	0.409
Gen. health → MHI	0.003	−0.177	0.225	0.126	0.984	0.023	−0.279	0.387	0.173	0.896
Income → MHI	0.110	−0.106	0.339	0.132	0.404	0.257	−0.004	0.603	0.156	0.098
Soc. cohesion → MHI	0.016	−0.133	0.162	0.080	0.842	−0.148	−0.542	0.171	0.176	0.401
**Indirect effects**										
Gen. health → PHI → MHI	−0.229	−0.422	−0.108	0.113	0.042	−0.209	−0.444	−0.062	0.100	0.036
Gen. health → Feel. prep. → MHI	−0.049	−0.134	0.001	0.037	0.184	−0.014	−0.104	0.061	0.041	0.735
Income → PHI → MHI	−0.139	−0.302	0.072	0.130	0.287	−0.069	−0.288	0.080	0.091	0.446
Income → Feel. prep. → MHI	−0.034	−0.111	0.039	0.044	0.447	−0.007	−0.095	0.057	0.038	0.845
Soc. cohes. → PHI → MHI	−0.084	−0.209	0.019	0.064	0.184	−0.043	−0.218	0.138	0.091	0.635
Soc. cohes. → Feel. prep. → MHI	−0.026	−0.076	0.017	0.025	0.290	0.042	−0.069	0.203	0.068	0.535

^1^*β* = standardized estimate; 95L = lower bound of the bootstrapped 95% *β* confidence interval; 95U = upper bound of the bootstrapped 95% *β* confidence interval; SE = standard error; *p* = *p*-value; PHI = physical health impacts; MHI = mental health impacts; CC = climate change.
